# Spontaneous Mouse Behavior in Presence of Dissonance and Acoustic Roughness

**DOI:** 10.3389/fnbeh.2020.588834

**Published:** 2020-10-08

**Authors:** Olivier Postal, Typhaine Dupont, Warren Bakay, Noémi Dominique, Christine Petit, Nicolas Michalski, Boris Gourévitch

**Affiliations:** ^1^Institut de l’Audition, Institut Pasteur, INSERM, Paris, France; ^2^Sorbonne Université, Collège Doctoral, Paris, France; ^3^Syndrome de Usher et Autres Atteintes Rétino-Cochléaires, Institut de la Vision, Paris, France; ^4^Collège de France, Paris, France; ^5^CNRS, Paris, France

**Keywords:** auditory roughness, auditory consonance, auditory dissonance, temporal envelope, envelope modulations, aversive sounds, mouse behavior

## Abstract

According to a novel hypothesis (Arnal et al., 2015, Current Biology 25:2051–2056), auditory roughness, or temporal envelope modulations between 30 and 150 Hz, are present in both natural and artificial human alarm signals, which boosts the detection of these alarms in various tasks. These results also shed new light on the unpleasantness of dissonant sounds to humans, which builds upon the high level of roughness present in such sounds. However, it is not clear whether this hypothesis also applies to other species, such as rodents. In particular, whether consonant/dissonant chords, and particularly whether auditory roughness, can trigger unpleasant sensations in mice remains unknown. Using an autonomous behavioral system, which allows the monitoring of mouse behavior over a period of weeks, we observed that C57Bl6J mice did not show any preference for consonant chords. In addition, we found that mice showed a preference for rough sounds over sounds having amplitude modulations in their temporal envelope outside the “rough” range. These results suggest that some emotional features carried by the acoustic temporal envelope are likely to be species-specific.

## Introduction

An easy way to catch the attention of a conspecific individual and ensure an optimal sensory-motor reaction is to increase sound intensity, by screaming or crying. These two communication signals are considered to be innate and shared across many species, particularly mammals (Newman, 2007; Lingle et al., 2012). They are usually termed “alarm calls” and are uttered in dangerous situations (like the presence of a predator) or by infants looking for adult caregivers (Zuberbühler, 2009).

In humans, recent studies show that alarm sounds present a common acoustic property: they are “rough” (Arnal et al., 2015, 2019). Acoustic roughness, which arises from amplitude modulation of the temporal envelope of sounds, with a modulation frequency between 30 and 150 Hz, typically elicits unpleasant sensations and increases attention (Fastl and Zwicker, 2007).

Hypothetically, roughness could be a common feature of “alarm calls” which would involve analogous neuronal responses across mammalian species (Newman, 2007; Lingle et al., 2012). Consistent with this hypothesis, despite the obvious species specificities (Gouzoules and Gouzoules, 2000), “alarm calls” of one species can be eavesdropped by another (Magrath et al., 2015), and mammals of a given species can respond to infant cries of several other species (Lingle and Riede, 2014). However, whether auditory roughness can substantiate unpleasant perceptions or even reactions in non-human mammals remains largely unexplored.

Auditory roughness is also often seen as contributing to dissonance according to Helmholtz’s theory (Helmholtz and Ellis, 1895; McDermott, 2012), as dissonant chords are rougher (i.e., they contain larger depth of amplitude modulation within the roughness frequency range) compared to consonant ones (Vassilakis, 2001). Here again, in contrast with humans, the preference for consonant sounds over dissonant ones of mammals is inconsistent (Fannin and Braud, 1971; McDermott and Hauser, 2004; Sugimoto et al., 2009; Chiandetti and Vallortigara, 2011; Koda et al., 2013) and unknown in mice.

We investigated the behavioral response of C57Bl6JRj mice to consonant and dissonant chords, and rough sounds in general. To do so, we used a fully automatic apparatus allowing continuous monitoring of individual mouse behavior, over weeks, in response to various sounds, with no need for any conditioning or human intervention.

## Materials and Methods

### Apparatus: Audiobox

We used C57BL/6JRj mice (20 females and 19 males) between 65 and 80 days old at the beginning of experiments.

**FIGURE 1:**
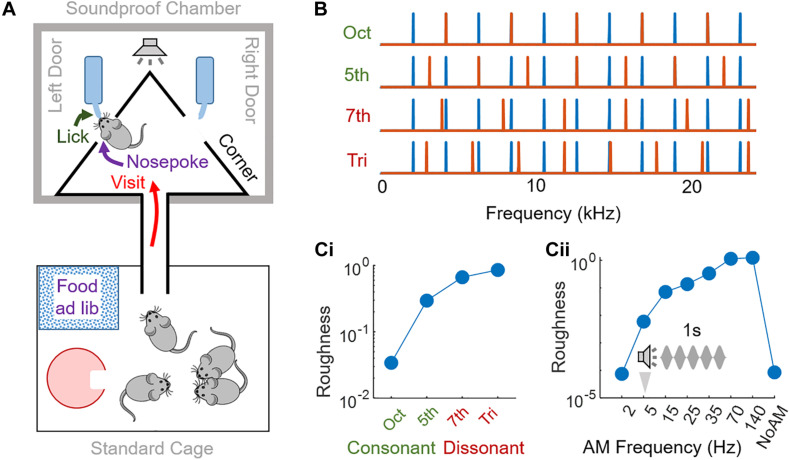
Experimental design. **(A)** Schematic of the Audiobox. The mice were kept in groups of 4–5 animals. A standard cage enriched with igloos served as the home cage, with food *ad libitum*, and was connected to a soundproof chamber through a corridor. The soundproof chamber contained the “corner,” the apparatus monitoring mouse behavior and controlling their access to water. A “visit” was registered when an antenna located at the entrance of the experimental corner read a transponder implanted in each mouse and a heat sensor was simultaneously activated. The mouse could then do a “nosepoke” into one of the two doors inside the corner and access water (“lick”). The stimulus was presented, through a speaker positioned 30 cm above the corner, either when a mouse visited or nosepoked, depending on the protocol, and according to the specific mouse that had been identified. **(B)** Spectrum of consonant and dissonant chords used. Frequency components of the first reference harmonic complex tone (blue) and the second tone (orange) were in ratios of (from top to bottom): 2 (octave, consonant), 3/2 (perfect fifth, consonant), 15/8 (major seventh, dissonant) or 45/32 (augmented fourth or tritone, dissonant). **(Ci,ii)** Roughness measure estimated thanks to (Vassilakis, 2001) for: **(Ci)** consonant and dissonant chords; **(Cii)** amplitude modulated reference harmonic complex tone.

As a behavioral monitoring system, we used the Audiobox (TSE systems, United States), which has been extensively described (de Hoz and Nelken, 2014) and the operation of which is summarized in Figure 1.

Each animal was individually identifiable by the Audiobox using a transponder (T-IS 8010 FDX-B, DATAMARS, Switzerland) implanted prior to behavioral testing in the upper back after a light anesthesia (Ketamine, 190 mg/kg; Xylazine 4.5 mg/kg; intraperitoneal). This reduced handling of the animals by the experimenter to the weekly cleaning of the cages and apparatus.

### Acoustic Stimuli

All sounds were generated using MATLAB software (Mathworks, United States) at a sampling frequency of 48 kHz. We created

— Consonant and dissonant chords by summing a reference note (C7, 2093 Hz) and its harmonics, all with equal amplitude, to another note, see Figure 1B. Chords were presented in sequences of 300 ms of sound separated by 100 ms silence.
— Amplitude Modulated (AM) sounds: broadband noise or a harmonic complex tone including 4 kHz and its harmonics, all with equal amplitude, were amplitude-modulated (100% of modulation depth) at rates of 2, 5 (see Figure 1Cii), 15, 25, 35, 70, and 140 Hz.

Sounds were presented at 77 (±2) dB SPL using a dome tweeter (22TAF/G, Seas prestige). Roughness of sounds was estimated using Vassilakis’ estimation method (2001) implemented in the free Matlab MIR toolbox. Original formula from Vassilakis for the roughness of two tones of frequencies *f*_1_,*f*_2_, and amplitudes *a*_1_,*a*_2_ is $r=\frac{{\left(a1a2\right)}^{0.1}}{2}{\left(\frac{2\min\left(a1,a2\right)}{a1+a2}\right)}^{3.11}Z$ where $Z=e^{-3.5\text{F}}-e^{-5.75\text{F}},F=S\left(\min\left(f_{1},f_{2}\right)\right)\left|f_{1}-f_{2}\right|$ and *S* = $\frac{0.24}{0.0207f+18.96}$. The roughness for more complex sounds (as here) is estimated by adding the roughness of all the possible individual tone-pairs.

### Protocols

#### Habituation

All our protocols began with a “transition” phase of 2–3 days (not shown in results), during which bottles of water were freely available in the home cage. In the following phase (“habituation,” 3–4 days), the bottles of water were then placed into the experimental corner. The doors were open all the time and no sound was emitted when a mouse was visiting or nosepoking. During these two phases, or during any following protocol, an animal was excluded from the experiment if its weight decreased by 20% or if it noticeably suffered from injury or stress.

#### Protocol 1, Passive Sound Listening

Each sound set consists of five possible sounds played. During each visit, one sound from the sound set is chosen randomly (probability 20%) and played until the mouse leaves the corner. The different sound sets successively tested (each over 3–6 days) were:

(1) Consonant/Dissonant Chords: Silence, octave chord (Oct), perfect fifth (5th), major seventh (7th), tritone (Tri).
(2) AM Noise Experiment 1: Silence, AM broadband noise with modulation frequencies (MFs) of 5, 15, 25, and 35 Hz.
(3) AM Noise Experiment 2: Silence, AM broadband noise with MFs of 2, 70, 140 Hz and no AM.
(4) AM Complex Tone Experiment 1: Silence, AM reference harmonic complex tone with MFs of 5, 15, 25, and 35 Hz.
(5) AM Complex Tone Experiment 2: Silence, AM reference harmonic complex tone with MFs of 2, 70, 140 Hz and no AM.

#### Protocol 2, Two-Choice

For 6 days, nosepoking was associated to the presentation of either sound 1 (a given door) or sound 2 (the other door). The preference index of one animal toward one sound or another was as follows:

PreferenceIndexnosepoking=nosepokesforsound 1nosepokesatsamedoorinsilence-nosepokesforsound 2nosepokesatsamedoorinsilencenosepokesforsound 1nosepokesatsamedoorinsilence+nosepokesforsound 2nosepokesatsamedoorinsilence

This preference index controls for the bias induced by the baseline preference to a given door, which was determined beforehand, in silence, during the habituation phase. The preference index ranges between −1 and 1, with the extremes showing nosepoking exclusively to the doors associated with sounds 2 or 1, respectively. A similar index was built from licking instead of nosepoking.

### Analysis and Statistics

Our data was analyzed using ANOVA tests with one factor (sound, dubbed ANOVA) or two factors (sex and sound, dubbed ANOVA 2) and *post-hoc t*-tests with Tukey-Kramer correction. To improve readability, for most tests used in the manuscript, we inserted a reference (a small letter in subscript) linking to the full details of the test in Supplementary Table 1.

## Results

### Mouse Behavior Is Insensitive to Consonant vs. Dissonant Sounds

**FIGURE 2:**
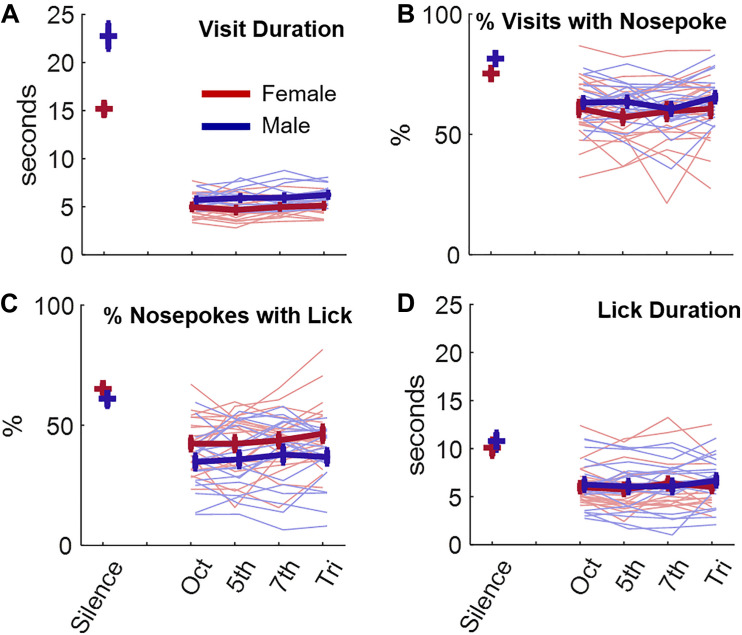
Effects of listening to consonant or dissonant chords on behavior. **(A)** Visit duration in seconds as a function of the chord presented or during silence. The behavior of female (red) and male (blue) animals is displayed. Thin lines are individual data while thick lines display average +/– standard error bars. **(B–D)** Same as **(A)** for the percentage of nosepoking during each visit **(B)**, for the percentage of licking during each nosepoke **(C)**, and for the lick duration **(D)**.

We first assessed the behavior of mice in silence and in the presence of consonant (octave, 5th) and dissonant (7th, tritone) chords. Mice made 148 ± 38.7 (STD) visits per day (Supplementary Figure 1A). Both male and female mice nosepoked and licked less, and durations of both visits and licks were shorter, when a sound was presented vs. when no sound was played (Figure 2, ANOVA 2, for all variables, sound effect^a1,a2,a3,a4^ *p* < 1e-10, *post-hoc* silence vs any sound^b1,b2,b3,b4^ *p* < 1e-3). Nonetheless, there was no effect of the chord heard at each visit on any subsequent visiting, nosepoking or licking behavior of the animal (ANOVA 2 without silence, all variables, sound effect^c1,c2,c3,c4^, *p* > 0.33). In addition, there was no effect of the chord heard on the first day either, suggesting that this result was not due to any habituation to the stimuli (Supplementary Figure 1B, ANOVA 2 without silence, all variables, sound effect^d1,d2,d3,d4^, *p* > 0.78). In general, the behavior was slightly different between males and females, with females visiting and nosepoking less but licking more for each nosepoke (ANOVA 2 factors sex and sound, sex effect, visit duration^d1^, % nosepoking^d2^, % licking^d3^ *p* < 5e-3, lick duration^d4^ *p* = 0.42, Figure 2).

Changing the way sounds were played, i.e., using continuous versions of chords instead of sequences of 300-ms long chords interspaced by 100-ms long silences, did not elicit a change of mice behavior in response to consonance and dissonance (ANOVA without silence, all variables^e1,e2,e3,e4^, *p* > 0.91, Supplementary Figure 1C). Increasing the sound level at 82 dB SPL instead of 77 dB SPL also resulted in no effect of consonance/dissonance on mouse behavior (ANOVA without silence, all variables, sound effect^f1,f2,f3,f4^, *p* > 0.2, Supplementary Figure 1D). These results suggest that mice were not sensitive to consonance or dissonance, at least not as defined by the literature and examined by our protocols. However, after the first day at 82 dB SPL (purple curve, Supplementary Figure 1D), there was a small trend toward a greater duration of both visits and licks during tritone and 7th chords (ANOVA without silence, visit duration, lick duration, sound effect^g1,g2,g3,g4^, *p* < 0.03, *post-hoc* Oct and 5th vs. Tritone for lick duration, *p* < 0.02). A preference for dissonant chords may have quickly vanished after a few days due to some habituation of the animals. We found this result intriguing but the trend was weak. One isolated parameter contributing to the pleasantness of consonant/dissonant sounds could have a stronger effect on animals. We decided to focus on roughness, which has long been suspected of partially explaining the aversive effect of dissonant chords.

### Mouse Behavior Is Highly Sensitive to the Roughness of Sounds

**FIGURE 3:**
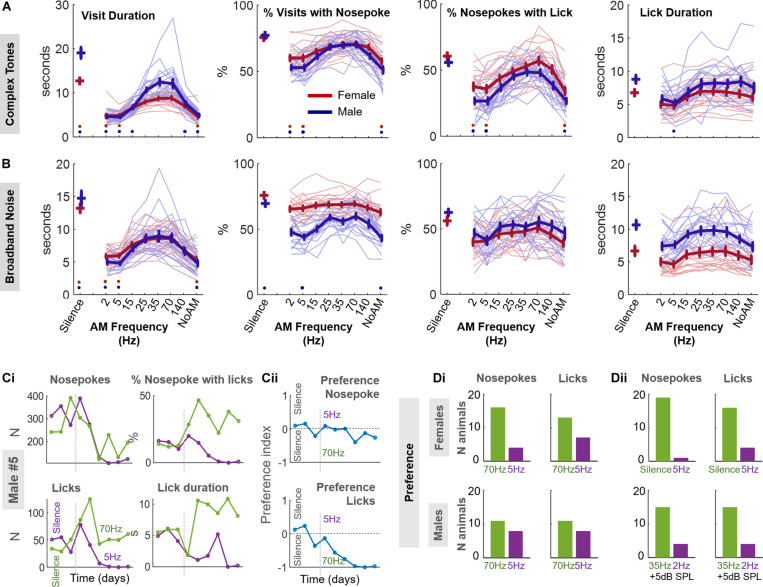
Effects of AM sounds on mouse behavior. **(A)** The carrier of the AM sound is a complex tone. From extreme left to right: visit duration, percentage of visits with a nosepoke, percentage of nosepokes with a lick, and lick duration, are represented as a function of the stimulus presented (complex tone or silence). The behavior of female (red) and male (blue) animals is displayed. Thin lines are individual data while thick lines display average +/− standard error bars. Red (resp. blue) points at a given AM rate indicate a *p* < 0.05 for the *post-hoc* test (see “Materials and Methods” for ANOVA definition) between this AM rate and the 35 Hz for female (resp. male) data. **(B)** Same as A when the carrier is broadband noise. **(C,D)** Two-choice protocol. After a 3-day habituation period, each door of the experimental corner is associated with an AM complex tone (5 Hz on left door, 70 Hz on right door) for 6 days. **(C)** Example results for male #5. **(Ci)** Number of nosepokes (upper left), licks (lower left), percentage of licking for each nosepoke (upper right) and lick duration (lower right) in each door across days. Green (resp. purple) line represents the right (resp. left) door. **(Cii)** Preference index for nosepokes (up) and licks (down) across days, see “Materials and Methods.” **(Di)** Numbers of animals which nosepoke (up) and lick (down) preferentially at the door associated with 70 or 5 Hz for females (top) or males (bottom). **(Dii)** Same than **(Di)** for females tested on the silence vs 5 Hz protocol (top) or males tested on the protocol 35 vs. 2 Hz with an additional 5 dB SPL for both sounds (bottom).

We scrutinized whether our animals exhibited a similar behavior in response to sounds with varying levels of roughness (Figures 3A,B). Using the same approach of passive listening as for the consonant/dissonant chords, we tested amplitude modulated sounds at 2, 5, 15, 25, 35, 70, and 140 Hz, the carrier sounds being either complex tones (fundamental frequency 4 kHz) or broadband noise (see section “Materials and Methods”). We also tested these carrier sounds with no amplitude modulation. This case sounds like, and is mathematically equivalent to, having an infinitely fast AM.

Unlike the consonant/dissonant chords, the varying AM rate of sounds elicited distinct behaviors which were robust across gender and stimuli (Figures 3A,B). For complex tones and for very slow AM rates (2 and 5 Hz) or no AM, the visits of the animals were typically shorter and the percentage of visits with a nosepoke and a lick was also smaller (complex tones, ANOVA 2^h1,h3,h4^, *post-hoc* 35 Hz vs 2, 5 Hz, no AM, *p* < 0.02) than for 25–70 Hz AM rates. This same pattern appeared in the presence of AM modulated broadband noise but the difference between 35 Hz and the other frequencies was significant only for visit duration (AM noise, ANOVA 2 factors sex and sound^i1^, *post-hoc* 35 Hz vs. 2, 5 Hz, no AM, *p* < 1e-4).

We could also see this pattern for both AM complex tones and broadband noise when counting the number of times the animal nosepoked during each visit (Supplementary Figures 2A,B). Overall, visit duration was the variable most strongly modulated by the AM rate, irrespective of the animal sex or the sound carrier used (Supplementary Figure 2C). We did not find any adaptation to the stimuli, as behavioral patterns were very similar on the first day to those observed throughout the protocol (Supplementary Figures 2D,E).

For both males and females, and for both types of stimuli, the duration of a visit was shorter when a sound was presented than when it was not (ANOVA 2, for either AM complex tones or broadband noise, sound effect^h1,i1^ *p* < 1e-10, *post-hoc* silence vs any AM rate *p* < 1e-4) consistent with our previous finding (Figure 2A). However, several AM rates in the roughness range elicited similar values as silence for lick duration, the percentage of visits with a nosepoke or the percentage of nosepokes with a lick (Figures 3A,B; ANOVA 2 factors sex and sound, for either AM complex tones or broadband noise, sound effect^h2,h3,h4,i2,i3,i4^ *p* < 2.8e-6, *post-hoc* silence vs 25, 35, and 70 Hz, *p* > 0.07). As for consonant/dissonant protocols, the females visited and nosepoked less but licked more for each nosepoke, with shorter durations, than males (ANOVA 2 factors sex and sound, sex effect^j1,j2,j3,j4,k2,k3,k4^, visit and lick duration, % visit with nosepoke, % nosepoke with lick *p* < 1.3e-4 except visit duration for females^k1^, *p* = 0.58).

### Mice Show Preference for Rough Sounds

We then wondered whether a shorter visit or lick duration could be interpreted as discomfort to the animal. To test this hypothesis, we designed a two-choice protocol where each of the two doors to which the animal nosepokes is associated to a particular sound. In a preliminary experiment, we tested female mice with the protocol contrasting the 5 Hz AM stimulus with silence. Since silence was associated with the longest visit and lick duration of all stimuli, we reasoned that we should observe a marked preference for silence compared to 5 Hz. Indeed, nineteen animals over twenty preferentially chose the door associated to silence after a few days (i.e., average preference index < 0 after the habituation period, see “Materials and Methods;” Figure 3Dii), giving confidence that mice can make a conditioned place preference based on presented sounds.

For both males and females, we then contrasted an AM rate putatively associated to discomfort (5 Hz, left door) and another AM rate associated with longer visit or lick durations (70 Hz, right door). As displayed in the individual example in Figures 3Ci,ii, after showing little preference for either of the two doors in the habituation period, male #5 progressively nosepoked and licked more and licked longer at the 70 Hz (right) door compared to the 5 Hz (left) door. Based on the preference index, there was a significant majority of animals who preferred 70 Hz AM sounds over 5 Hz AM sounds for nosepokes but not for licks (Figure 3Di; proportion test vs 50%, pooled genders: nosepokes, stat = 2.49, *p* = 1.6e-2; licks, 1.44, *p* = 0.15). This preference was more pronounced for female mice. Raw data for nosepokes, licks and preference index shows that this preference did not vanish, but rather stabilized or increased with days, suggesting that mice did not adapt to the discomfort or progressively tended to reproduce their previous behavior (Supplementary Figures 3A–C). In males, we obtained an increased preference (15 animals vs. 4) to rough sounds by contrasting the rough 35 Hz AM sounds to 2 Hz AM sounds and increasing the SPL by 5 dB (Figure 3Dii). Overall, these results suggest that rough sounds with AM frequencies of 35/70 Hz were preferred by a majority of animals over sounds with slower AM frequencies of 2/5 Hz.

## Discussion

Our study examined whether so-called consonant and dissonant chords, and more generally rough sounds, hold the same aversiveness for laboratory mice as they do for humans. To begin, the duration of visits was always greater during silence compared to any sound stimulus (dissonant/consonant chords, AM sounds or non-AM sounds), for both male and female mice. Consistently, previous studies showed that mice would have an innate bias for silent shelter over music or pure tones (Jouhaneau and Bagady, 1984; Yang et al., 2012). We interpret silence as the most pleasant, or least stressful, situation for the mouse, suggesting that sounds are generally unpleasant or stressful for mice.

We then found that mice did not show any behavioral difference, and therefore putatively any sign of additional discomfort, in response to consonant or dissonant chords. It is possible that mice did not discriminate between consonant and dissonant chords. However, this hypothesis is unlikely because rats can discriminate between consonant and dissonant chords (Crespo-Bojorque and Toro, 2015) and mice have excellent frequency resolution between 6 and 15 kHz (de Hoz and Nelken, 2014). Our passive listening protocol might not have been able to show mouse preference to either consonant or dissonant sounds. However, this protocol was highly effective in revealing the sensitivity of mice to roughness. We are therefore confident that the mice did not show any preference for the consonant and dissonant chords we used. In general, consonance/dissonance perception and emotion have been poorly explored outside of humans, with inconsistent results. Chicks seem to prefer consonant chords over dissonant ones (Chiandetti and Vallortigara, 2011) while monkeys do not (McDermott and Hauser, 2004; Koda et al., 2013) but infant apes do (Sugimoto et al., 2009). Rats prefer consonant chords (Fannin and Braud, 1971; Borchgrevink, 1975) but concerns about the stimuli used were raised (Rogers, 2010). In fact, if there was any preference for one type of chord in mice, it could be for dissonant chords, at least on the first day of sound presentations, and at increased SPL (Supplementary Figure 1D).

Intrigued by this intermediate result, we next focused on the acoustic roughness as one isolated acoustic feature of dissonant sounds. Mice showed shorter visits and licks for AM sounds at rates 2–5 Hz or with unmodulated sounds, and longer ones for AM sounds in the 25–70 Hz range, which corresponds to the lower part of the acoustic roughness range. This pattern applied to all behavior parameters we measured, for both noise and complex tone carriers, but was typically more prominent for complex tones. According to Morton motivation-structural rules, tonal high-frequency natural sounds produced by mammals and birds should be associated with friendly situations, whereas more broadband, low-frequency sounds should be associated with hostile situations (Morton, 1977). This hypothesis is not supported by our data: complex tones elicited shorter visit durations than broadband noise for AM rates of 2, 5, and 15 Hz, and no longer durations for other AM rates or for unmodulated noise. We did not observe that animals familiarized, or habituated, to our sounds as patterns of visit duration were very stable over time in the passive listening protocol and the preference to a sound stabilized or increased with time in the two choice protocol. As a possible explanation for this last result, mice could have preferred following the same behavior as they had previously, especially in a colony where all animals including the dominant one preferentially chose one door.

Females tended to visit the experimental corners less frequently, but licked more than males for a given visit. More importantly, AM rates affected the behavior more strongly in males than females (Supplementary Figure 2C). This could be related to sexual differences in sound perception as, for instance, response to pup or adult mice vocalizations differ between males and females (Ehret et al., 1987; Hammerschmidt et al., 2012). Sexual differences at the circuit level could also be involved. For example, recent work showed a sexual dimorphic distribution of cannabinoid receptor mRNA in the brains of C57BL/6J mice (Liu et al., 2020), a receptor that is linked to emotional modulation and anxiety-like behavior in rodents and humans (Akirav, 2011; Bowers and Ressler, 2016), two features which influence sensory perception.

In the two-choice protocol, a majority of mice, irrespective the sex, preferentially chose the door associated with the rough AM sound (70 Hz) for nosepoking. These results and those from the passive listening protocol could be interpreted both as a preference for rough sounds or an avoidance or unpleasantness of low and fast AM rates. The hypothesis that rough AM sounds are considered pleasant for mice, or at least as neutral as silence, is strikingly different to human results. In humans, rough sounds are associated with unpleasantness, with the strongest effect around 70 Hz (Fastl and Zwicker, 2007), and trigger neural pathways associated with aversive perception and defensive behaviors (Arnal et al., 2015, 2019). For instance, a baby’s cries are one of the roughest sounds that humans can produce, and they elicit an extremely unpleasant sensation (Li et al., 2018). Where does the difference between mice and humans come from? In humans, roughness occupies an acoustic niche between the slow amplitude modulations associated to the syllabic rate and the low temporal envelope (<30 Hz) and the faster ones (>100 Hz), which elicit a perception of pitch. These features are highly human related and it is not likely that an equivalent range exists for mice. Moreover, albeit rough, dissonant chords elicited parameter values (e.g., visit and lick duration) generally closer to those observed for 2–5 Hz than for 70 Hz: i.e., they would be associated with rather unpleasant sounds. Overall, consonant and dissonant chords did not modify mouse behavior. Thus, we cannot exclude that roughness as we computed it (based on observed dissonance in humans, Vassilakis, 2005) is not a salient acoustic component for mice. For instance, mice could have been sensitive to the slope of the ramp in each sinusoidal cycle of AM sounds (Deneux et al., 2016). However, in other mammals (otters, primates or bats), studies indicate that fast AM sounds, such as a baby’s cry, are also produced in alarm calls, and/or antagonistic interactions (Leinonen et al., 2003; Mumm and Knörnschild, 2017; Hechavarría et al., 2019). Since infant distress calls appear to share acoustic similarities and analogous neuronal responses across mammals (Newman, 2007; Zuberbühler, 2009; Lingle et al., 2012; Lingle and Riede, 2014), it is often suggested that rough sounds could elicit unpleasantness in many species as well. Consistently, it was shown that roughness can be aversive to seals (Götz and Janik, 2010). We show here that at least one species, mouse, perceives rough sounds as not unpleasant.

The hypothesis that both slow and fast AM rates could be unpleasant for mice is particularly unclear and novel. Shorter visit and lick duration can indeed be interpreted as an avoidance. It has been shown that both natural and artificial aversive sounds can disturb animals such that they eat less or escape the area (Biedenweg et al., 2011). Avoidance is a classical correlate of using aversive sounds in conditioning (Mackintosh, 1983). In our study, only a short majority of mice, irrespective the sex, preferentially chose the door associated with the rough AM sound (70 Hz) for nosepoking in the two-choice protocol. Thus, it is likely that low and fast AM rates are unpleasant rather than strongly aversive. What are the reasons behind such unpleasantness? Spectro-temporal processing circuits in mice may have co-evolved with their conspecific vocalization processing (Ehret, 2001; Green et al., 2019; Cai and Dent, 2020). From that perspective, slow AM rates around 2–5 Hz could interfere with the emitting rate of vocalization in mice (see e.g., sequences of ultrasonic vocalizations in Fischer and Hammerschmidt, 2011; Castellucci et al., 2018). That such signals are not comprehensible to the mice, but could potentially be vocalized from other mice, might elicit anxiety in these animals.

**FIGURE 4:**
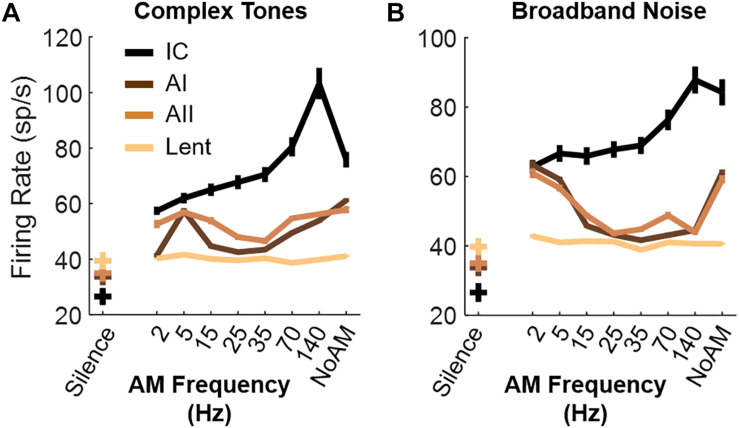
Multiunit recordings of auditory (AI, AII, IC) and non-auditory (Lent) neurons. **(A,B)** Average firing rate in response to AM complex tones **(A)** and AM broadband noise **(B)**. Lines display average +- standard errors. *N* = 8 C57BL/6JRj female mice, 5–8 weeks old (JANVIER Labs). Brief summary of the protocol: surgery of mice (craniotomy) was done under Ketamine (190 mg/kg) and Xylazine (4.5 mg/kg) anesthesia. Recordings were done under Isoflurane (0.8–1% isoflurane in a flow rate of 0.2 L/min of 95% O_2_) anesthesia 1 h after the IP injection. Using the stereotaxic coordinates (Paxinos and Franklin, 2019) and knowledge of vasculature, a matrix of 4 electrodes each containing 8 recording sites (Buzsaki32-CM32, Neuronexus, United States, cortical structures) or 32-channel laminar electrode (NeuroNexus, subcortical structures) was implanted in the primary auditory cortex (AI), the secondary auditory cortex (AII), the central nucleus of the Inferior Colliculus (IC) and the Lateral part of the entorhinal cortex (Lent). The high frequency signal (0.3–10 k Hz) acquired by the electrophysiology system (OmniPlex, Plexon Inc., United States) contained the extracellular action potentials of neurons. In AI and AII, the recording depth was 400–700 μm, corresponding mostly to the layer V of the cortex (Chang and Kawai, 2018). In all areas, responses to 30 repetitions of AM noise and AM complex tones (for each AM rate) or 180 s of silence were recorded.

It is possible that the preference of mice for silence, or between AM rates, also relies on plasticity mechanisms of their neural circuits: the preference of mice for specific types of music can be forced by early auditory exposure during the critical period (Jouhaneau and Bagady, 1984; Yang et al., 2012). For instance, mice could prefer silence simply because they grew up in a silent environment (Yang et al., 2012). Another explanation could lie in the neural response of the auditory pathways. Several studies have noticed that the firing rate in the auditory cortex of other mammals typically decreases when the modulation rate reaches 10–20 Hz before increasing again when the modulation rates exceeds 100–200 Hz (Schreiner and Urbas, 1988; Schulze and Langner, 1997; Walton et al., 2002; Joris et al., 2004; Gourévitch and Eggermont, 2010). As part of another project, we recorded the firing rate of the same strain of mice to our AM stimuli and confirmed this low firing rate in the 15–70 Hz range in the primary and secondary auditory cortices (Figure 4). Thus, the reduced firing rate in the auditory cortex when AM is within the roughness range could be a neural correlate of a weak sensory stimulation, an unstressed emotional state or more simply a lower perceived loudness by the animals. This explanation would be consistent with the smaller level of activity under silent conditions compared to stimuli presentation (Figure 4).

However, similar variations of firing rate with AM or silence are likely to occur in humans as well and would therefore not explain the contrast of pleasantness between humans and mice. A broader hypothesis could be that the neural circuits linked to sound euphony might be completely different in mice and humans. Arnal et al. (2019) suggest that the aversive sensation induced by rough sounds results from “the persisting, exogenous synchronization of large-scale (limbic) networks involved in salience rather than specifically auditory – processing.” In mice, Zhang and colleagues identified a non-canonical reticular-Limbic central auditory pathway associated with fear conditioning (Zhang et al., 2018b) involving structures such as the medial septum, and a septal-habenular pathway processing aversive emotion (Zhang et al., 2018a). Future electrophysiology experiments should explore neuronal responses of mice to AM sounds in brain areas related to the limbic system.

## Conclusion

We show that mice do not prefer consonant over dissonant chords, at least under passive listening conditions. Moreover, in contrast with humans, mice seem to perceive rough sounds as pleasant and temporal envelope modulations around 2–5 Hz, a range of important speech features in humans (such as the syllabic rate), as unpleasant. Although their mechanisms remain unclear, our results question the validity of roughness as a common feature of aversiveness among mammals. Further, visit duration to an area associated with sounds might be a relevant parameter to compare the pleasantness of such sounds without having to condition the animals.

## Data Availability Statement

The raw data supporting the conclusions of this article will be made available by the authors, without undue reservation.

## Ethics Statement

The animal study was reviewed and approved by the Institut Pasteur Ethics Committee for Animal Experimentation.

## Author Contributions

BG designed the research. BG, OP, and TD analyzed the data. BG, OP, TD, WB, and NM wrote the manuscript. BG, OP, TD, WB, and ND performed the research. NM and CP provided the resources. All authors revised and approved the final version of the manuscript.

## Conflict of Interest

The authors declare that the research was conducted in the absence of any commercial or financial relationships that could be construed as a potential conflict of interest.
