# Supplementary material for: Spontaneous Mouse Behavior in Presence of Dissonance and Acoustic Roughness
Source: Front Behav Neurosci. 2020 Oct 8;14:588834. doi: 10.3389/fnbeh.2020.588834 (PMC7578920; doi:10.3389/fnbeh.2020.588834)
Supplement: Supplementary file 1 [file Table_1.DOCX]

## **Supplementary Table**

| Reference index | Test description | Statistics | P value |
| --- | --- | --- | --- |
| a1 | ANOVA 2 factors sex and sound with interaction, visit duration, sound effect | F(4,185)=283 | <1e-10 |
| a2 | ANOVA 2 factors sex and sound with interaction, lick duration, sound effect | F(4,185)=18.4 | <1e-10 |
| a3 | ANOVA 2 factors sex and sound with interaction, % nosepoking, sound effect | F(4,185)=21.8 | <1e-10 |
| a4 | ANOVA 2 factors sex and sound with interaction, % licking, sound effect | F(4,185)=26.1 | <1e-10 |
| b1 | Post-hoc t-test with Tukey-Kramer correction, silence vs any AM frequency |  | <1e-4 |
| b2 | Post-hoc t-test with Tukey-Kramer correction, silence vs any AM frequency |  | <1e-3 |
| b3 | Post-hoc t-test with Tukey-Kramer correction, silence vs any AM frequency |  | <4e-4 |
| b4 | Post-hoc t-test with Tukey-Kramer correction, silence vs any AM frequency |  | <2e-4 |
| c1 | ANOVA 2 factors sex and sound with interaction, silence not included, visit duration, sound effect | F(3,148)=1.15 | 0.33 |
| c2 | ANOVA 2 factors sex and sound with interaction, silence not included, lick duration, sound effect | F(3,148)=0.25 | 0.86 |
| c3 | ANOVA 2 factors sex and sound with interaction, silence not included, % nosepoking, sound effect | F(3,148)=0.64 | 0.59 |
| c4 | ANOVA 2 factors sex and sound with interaction, silence not included, % licking, sound effect | F(3,148)=0.49 | 0.69 |
| d1 | ANOVA 2 factors sex and sound with interaction, visit duration, sex effect | F(1,185)=52 | <1e-10 |
| d2 | ANOVA 2 factors sex and sound with interaction, % nosepoking, sex effect | F(1,185)=8 | 5e-3 |
| d3 | ANOVA 2 factors sex and sound with interaction, % licking, sex effect | F(1,185)=13.8 | 2.7e-4 |
| d4 | ANOVA 2 factors sex and sound with interaction, lick duration, sex effect | F(1,185)=0.64 | 0.42 |
| e1 | ANOVA 1 factor sound, silence not included, visit duration | F(3,56)=0.1 | 0.96 |
| e2 | ANOVA 1 factor sound, silence not included, lick duration | F(3, 56)=4e-3 | 1 |
| e3 | ANOVA 1 factor sound, silence not included, % nosepoking | F(3, 56)=0.18 | 0.91 |
| e4 | ANOVA 1 factor sound, silence not included, % licking | F(3, 56)=0.18 | 0.91 |
| f1 | ANOVA 1 factor sound, silence not included, visit duration | F(3,72)=1.59 | 0.2 |
| f2 | ANOVA 1 factor sound, silence not included, lick duration | F(3, 72)=1.5 | 0.22 |
| f3 | ANOVA 1 factor sound, silence not included, % nosepoking | F(3, 72)=0.2 | 0.9 |
| f4 | ANOVA 1 factor sound, silence not included, % licking | F(3, 72)=0.58 | 0.63 |
| g1 | ANOVA 1 factor sound, silence not included, visit duration | F(3,72)=3.19 | 0.03 |
| g2 | ANOVA 1 factor sound, silence not included, lick duration | F(3, 72)=4.4 | 7e-3 |
| g3 | ANOVA 1 factor sound, silence not included, % nosepoking | F(3, 72)=0.37 | 0.77 |
| g4 | ANOVA 1 factor sound, silence not included, % licking | F(3, 72)=2.28 | 0.09 |
| h1 | ANOVA 2 factors sex and sound with interaction, AM complex tones, visit duration, sound effect | F(8,333)=106 | <1e-10 |
| h2 | ANOVA 2 factors sex and sound with interaction, AM complex tones, lick duration, sound effect | F(8,333)=7.31 | <1e-10 |
| h3 | ANOVA 2 factors sex and sound with interaction, % AM complex tones, nosepoking, sound effect | F(8,333)=34.4 | <1e-10 |
| h4 | ANOVA 2 factors sex and sound with interaction, % AM complex tones, licking, sound effect | F(8,333)=31.7 | <1e-10 |
| i1 | ANOVA 2 factors sex and sound with interaction, AM broadband noise, visit duration, sound effect | F(8,333)=58.5 | <1e-10 |
| i2 | ANOVA 2 factors sex and sound with interaction, AM broadband noise, lick duration, sound effect | F(8,333)=5.3 | 2.8e-6 |
| i3 | ANOVA 2 factors sex and sound with interaction, AM broadband noise, % nosepoking, sound effect | F(8,333)=18.4 | <1e-10 |
| i4 | ANOVA 2 factors sex and sound with interaction, AM broadband noise, % licking, sound effect | F(8,333)=7.5 | 3e-9 |
| j1 | ANOVA 2 factors sex and sound with interaction, AM complex tones, visit duration, sex effect | F(1,333)=59 | <1e-10 |
| j2 | ANOVA 2 factors sex and sound with interaction, AM complex tones, lick duration, sex effect | F(1,333)=23.7 | 1.7e-6 |
| j3 | ANOVA 2 factors sex and sound with interaction, % AM complex tones, nosepoking, sex effect | F(1,333)=15.4 | 1e-4 |
| j4 | ANOVA 2 factors sex and sound with interaction, % complex tones, licking, sex effect | F(1,333)=40 | 8e-10 |
| k1 | ANOVA 2 factors sex and sound with interaction, AM broadband noise, visit duration, sex effect | F(1,333)=0.3 | 0.58 |
| k2 | ANOVA 2 factors sex and sound with interaction, AM broadband noise, lick duration, sex effect | F(1,333)=109 | <1e-10 |
| k3 | ANOVA 2 factors sex and sound with interaction, % AM broadband noise, nosepoking, sex effect | F(1,333)=236 | <1e-10 |
| k4 | ANOVA 2 factors sex and sound with interaction, % broadband noise, licking, sex effect | F(1,333)=15 | 1.3e-4 |

Supplementary Table 1: details on statistical tests used in the manuscript

## **Supplementary Figures**


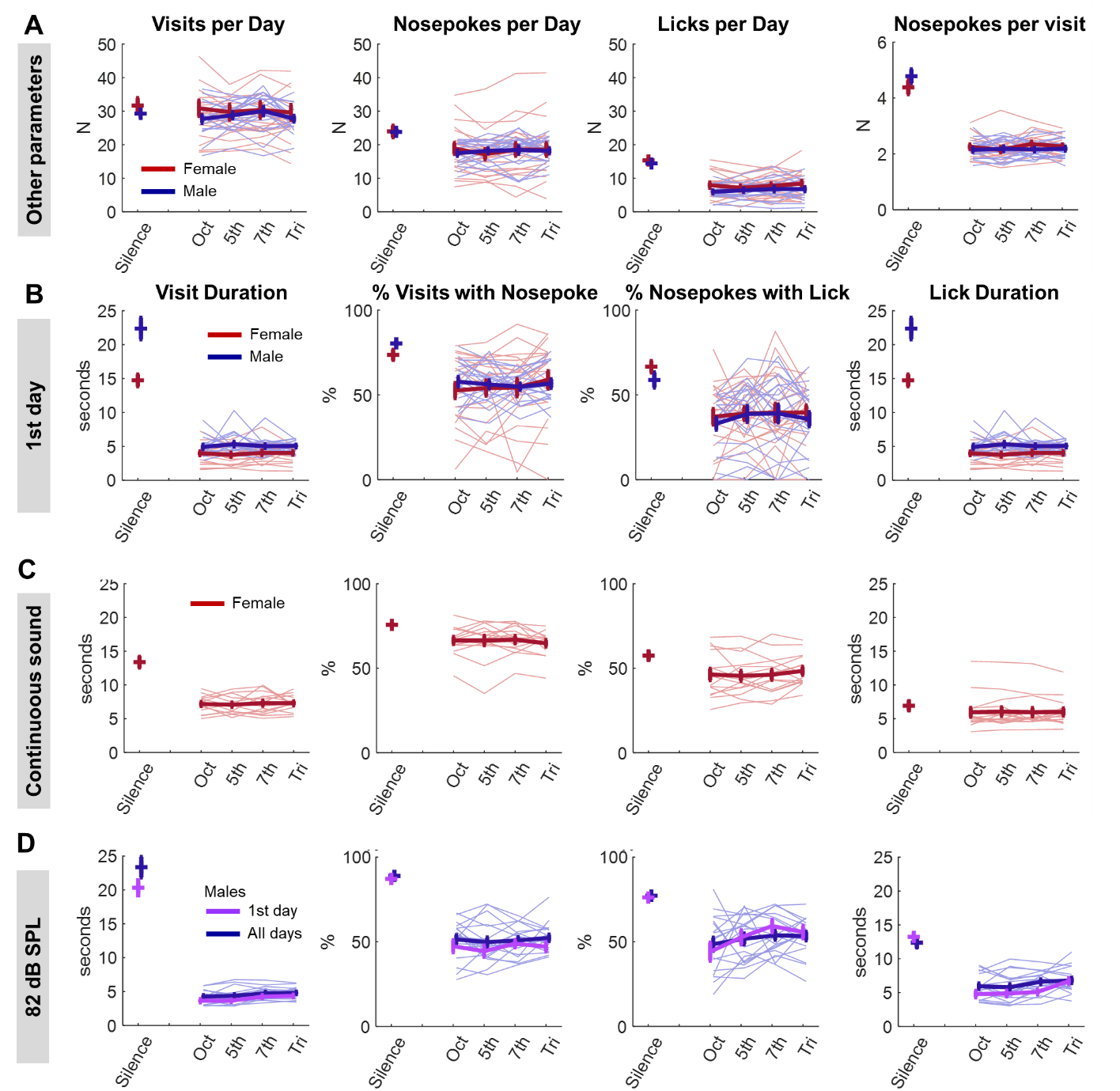


Supplementary Figure 1: Effects of listening to consonant (Octave, Perfect Fifth) or dissonant (Minor Seventh, Augmented Fourth i.e. Tritone) chords on behavior. A) From left to right: visits per day, nosespokes per day, licks per day and number of nosespoke per visit are shown as a function of the chord presented or during silence. Behavior of female (red) and male (blue) animals is shown, with thin lines indicating individual data while thick lines display average +- standard error bars, within subject. B) Same data as Fig. 2 but only including the first day. From left to right: visit duration, percentage of visits with a nosepoke, percentage of nosepokes with a lick and lick duration are represented as a function of the chord presented or during silence. C) Continuous sounds were presented instead of the short sound sequences used in Fig. 2. Only females were tested, with thin lines indicating individual data while thick lines display average +/- standard error bars. From left to right: same as in B). D) Sounds are presented at 82 dB SPL instead of 77 dB SPL as in Fig. 2. Only males were tested. From left to right: same as in B). Results for the first day (purple) and for all days (blue) are displayed. A,B,C,D) Thin lines are individual data while thick lines display average +- standard error bars.


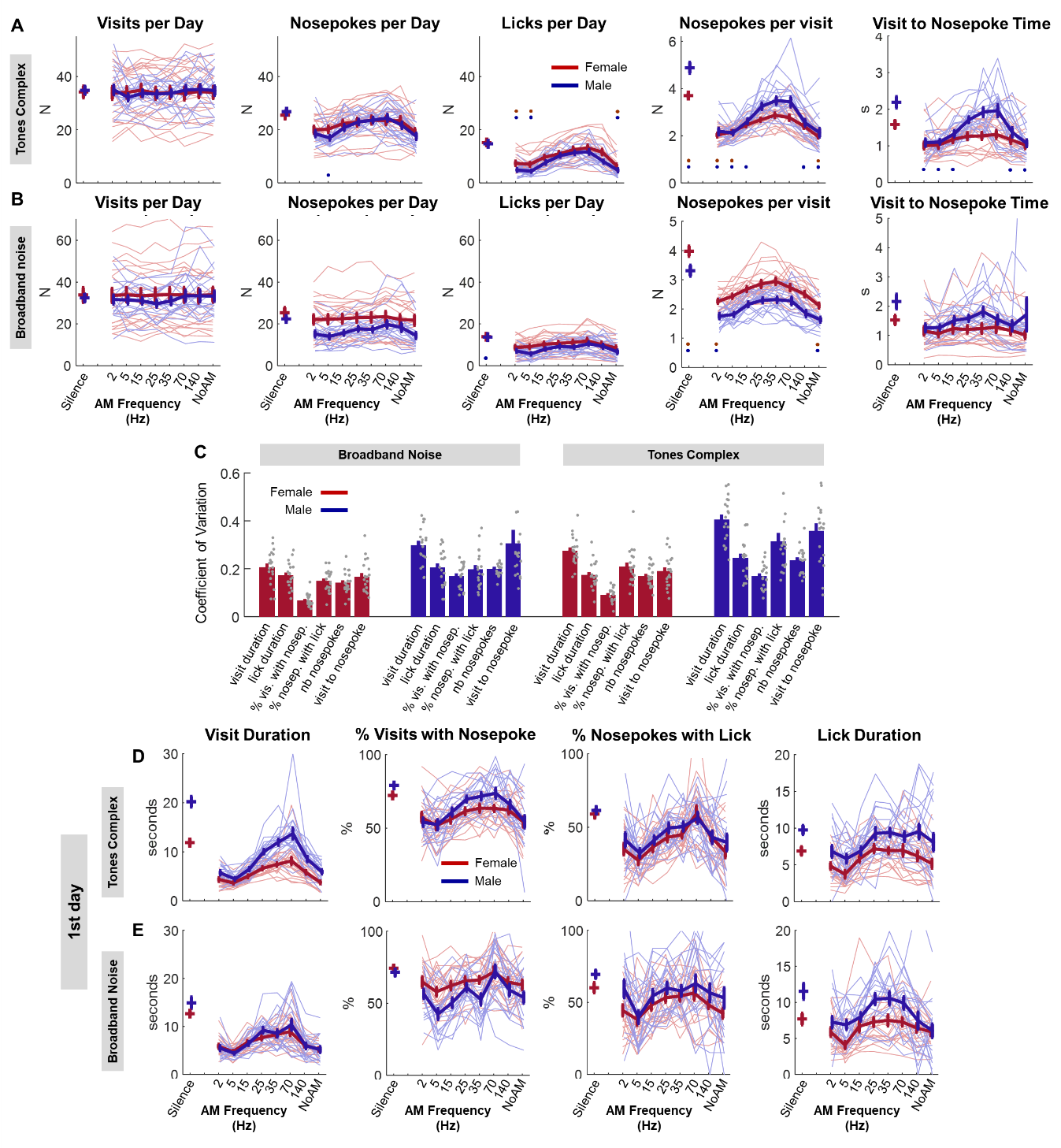


Supplementary Figure 2: Effects of AM sounds on behavior. A) The carrier of the AM sound is a complex tone. From extreme left to right: visit per day, nosepokes per day, licks per day, number of nosepokes per visit and visit to nosepoke time are represented as a function of the AM rate presented or during silence. The behavior of female (red) and male (blue) animals is shown, with thin lines indicating individual data while thick lines display average +- standard error bars. Red (resp. blue) points at a given AM frequency indicate a p<0.05 for the post-hoc test (see body text for ANOVA definition) between this AM frequency and the 35Hz for female (resp. male) data. B) Same as for A but the carrier is broadband noise. C) Coefficient of variation (ratio of standard deviation to the mean) for all parameters extracted from mouse behavior. Grey points are animals. Bars represent average + standard error. D,E) Same as Fig. 3A,B, respectively, but only including data from the first day.


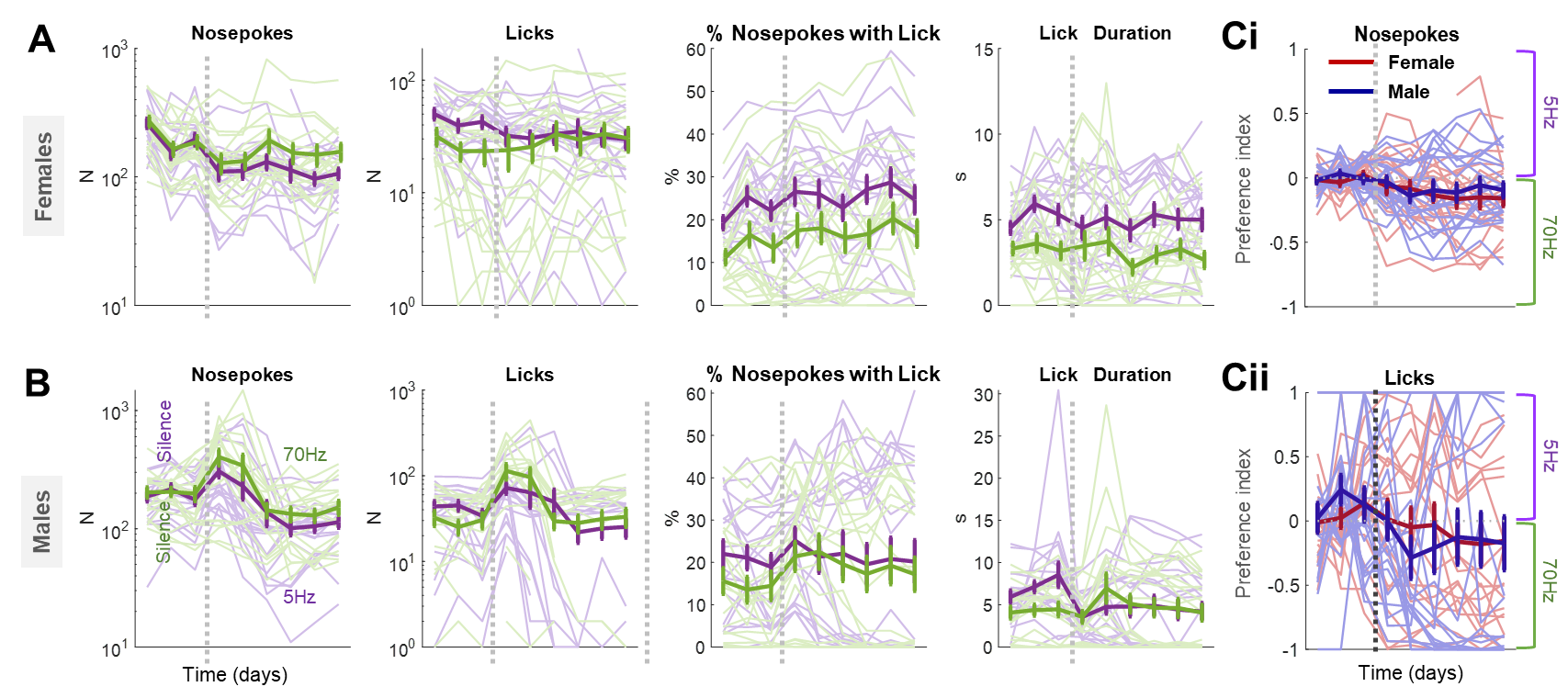


Supplementary Figure 3: Two-choice protocol. A-B) Group data for females (A) and males (B) when contrasting 5Hz and 70Hz. From left to right: numbers of nosepokes, licks, percentage of nosepokes with a lick, and lick duration in each door as a function of days on the abscissa. Green (resp. purple) line represents the right (resp. left) door of the experimental corner. Ci,Cii) Mean preference index (see methods) for nosepokes (Ci) and licks (Cii) across days. A preference index >0 (resp. <0) is associated to more nosepokes or licks to the door with an AM rate of 5Hz (resp. 70Hz). A,B,Ci,Cii) Thin lines are individual data while thick lines display average +- standard error bars.
